# Homestay Hosting Dynamics and Refugee Well-Being: Protocol for a Scoping Review

**DOI:** 10.2196/56242

**Published:** 2024-03-19

**Authors:** Areej Al-Hamad, Yasin M Yasin, Kateryna Metersky, Sepali Guruge, Khadija Mahsud

**Affiliations:** 1 Toronto Metropolitan University Toronto, ON Canada; 2 University of Doha for Science and Technology Doha Qatar

**Keywords:** cohabitation, homestay, hospitality, host family, host-guest relationship, refugee, well-being

## Abstract

**Background:**

The process of refugee resettlement and integration into new communities is a complex and multifaceted challenge, not only for the refugees themselves but also for the host families involved in homestay housing arrangements. While these homestay arrangements are designed to facilitate smoother transitions and enhance the well-being of refugees, the nuanced dynamics of these interactions and their overall impact on both refugees and their host families remain underexplored. Understanding the experiences of refugees and their host families is vital for effective refugee settlement, integration, and well-being. However, the intricacies of homestay refugee hosting, their interactions with host families, and the impact on their well-being are still unclear and ambiguous.

**Objective:**

The aim of this scoping review is to examine the breadth of literature on the experiences of refugees living in homestay arrangements with their host families. This review seeks to understand how these dynamics influence refugee well-being, including their integration, social connections, and mental health. Additionally, this scoping review aims to synthesize existing literature on homestay hosting dynamics, focusing on the experiences of refugees and their host families, to identify gaps in knowledge and suggest areas for future research.

**Methods:**

This scoping review follows Joanna Briggs Institute methodology and will search databases such as CINAHL, SOCIndex, MEDLINE through EBSCO; APA PsycInfo, Scopus through OVID; and Web of Science Core Collection, ProQuest Dissertations, and Theses, and SciELO Citation Index, focusing on literature from 2011 onward, in English, in relation to refugee groups in different host countries, including all types of literature. Literature will be screened by 2 independent reviewers, with disagreements resolved by consensus or a third reviewer. A custom data extraction tool will be created by the research team.

**Results:**

The results will be organized in tables or diagrams, accompanied by a narrative overview, emphasizing the main synthesized findings related to the dynamics of homestay hosting with host families and refugee well-being. No critical appraisal will be conducted. This scoping review is expected to identify research gaps that will inform the development of homestay refugee hosting models, policies, and practices. It will also offer insights into best practices and policy recommendations to improve homestay hosting programs, ultimately contributing to more effective refugee settlement and integration strategies.

**Conclusions:**

Understanding the intricate dynamics of homestay hosting arrangements is crucial for developing policies and programs that support the well-being of refugees and the families that host them. This scoping review will shed light on the current knowledge landscape, identify research gaps, and suggest ways to enhance the homestay hosting experience for all parties involved. Through this work, we aim to contribute to the development of more inclusive, supportive, and effective approaches to refugee hosting, resettlement, and integration.

**International Registered Report Identifier (IRRID):**

DERR1-10.2196/56242

## Introduction

### Overview

The global displacement of refugees amounted to 35.3 million individuals, with a majority comprising female individuals [1]. However, as the number of individuals seeking refuge in host countries grows, the housing needs of such people beckon resolution. One prosperous solution that has arisen from housing needs is refugee hosting [2]. Within the purview of refugee studies, the term “hosting” signifies the established societies or communities that undertake the resettlement of refugees [3]. Hosting communities are often credited with the expectation to foster welcoming and accepting environments for refugee women and their families to settle after escaping conflict [4].

Families seeking support may be placed, by publicly or privately funded organizations, into the homes of host families. Such homes are volunteered by willing families or individuals, many of whom are refugees or immigrants [4]. Hosting communities and their members often play a crucial role in extending hospitality and support to refugee women and their families to facilitate integration [5]. This culture of hospitality is also observed at the individual level notably within urban communities, where there is a rising trend of individuals and families hosting refugees in their private residences [6]. The role of these host families typically involves temporarily sharing their homes and resources with newly arrived refugees, all with the aim of enhancing the settlement process [6]. However, the use of the terms “host” and “hosting” illustrates a host-guest relationship, positioning refugees within the domain of the hosts. This conveys an underlying power imbalance between the hosts and the refugees [4,7]. This dynamic can also cultivate certain expectations imposed on refugees, dictating their behavior according to the host’s perceived standards of being worthy of hospitality [7]. For example, in a study by Monforte et al [7], exploring the responsibility of hosting, private hosts expressed emotional connection and mutual affection as a jointly shared responsibility with refugees, placing expectations on refugees to reciprocate. When refugees did not meet their expectations due to language barriers or unwillingness to discuss their past, hosts’ perception of the refugee’s value altered [7].

The expectation of the refugee to be a “good guest” highlights the conditional nature of private hosting, therefore requiring refugees to prove they are deserving of being hosted [4,5,7]. When refugees do not match the expectations or ideas of hosts, this can create an uncomfortable environment for refugees [5]. Furthermore, hosting arrangements and expectations can limit refugees’ control over their surroundings, as they are essentially guests in someone else’s domain [4,6,7]. This lack of control over their living situation can potentially hinder their ability to rebuild their lives autonomously, trigger power differences, and add an extra layer of vulnerability [8,9]. This power difference is even more pronounced for refugee women due to the intersection of gender-related disparities [10]. In addition, refugees' lack of host country citizenship further positions them as guests and makes them vulnerable to various abuses of power [11]. Viewing refugees women as mere guests within host homes and communities increases their social and economic vulnerabilities, especially without sufficient system-level policies and safeguards in place to protect their welfare [12].

Dağtaş [13] argues that refugee lived experience is heavily influenced by the host country’s political conditions and social welfare policies (for refugees). In addition, current literature has revealed that hosting frameworks have created a racialized discourse of nonbelonging [14]. Furthermore, reviews of the literature on current homestay or family hosting practices and regulations for refugee groups highlight an enormous gap [6]. Although communities and host families play an immense role in refugee settlement, refugee well-being relies heavily on standards being shaped by inclusive and informed social welfare policies and practices [15]. Host families and the communities in which refugees are hosted are pivotal in their successful settlement and success [7,15]. They may provide crucial aid to refugees, including more tangible forms of support, such as housing or financial aid, in addition to more abstract forms of assistance, such as cultural orientation, emotional support, and social comfort [7]. However, as generally unregulated social groups, the success of such support provided to refugees can vary largely based on cultural competency, empathetic disposition and understanding, and the attitudes and perspectives of communities and host families [4,15]. For a meaningful exploration of the hosting practices and dynamics implemented by the host families, it is imperative to understand the discourse these practices bring forth and their implications on refugees’ well-being.

A preliminary search of the literature through review registries (Open Science Framework and Joanna Briggs Institute (JBI) Evidence Synthesis) was conducted. No current or in-progress scoping reviews on this topic were identified. Existing reviews do not study the specific relationship between refugees and host families; reviews on the topic of refugee integration, in general, tend to be focused on a particular geography in terms of the host country or on a particular subset of refugees (eg, by gender, host country, or accommodations practices). The aim of this scoping review is to explore the experiences of refugees with their host families and understand the dynamics of homestay hosting on refugee well-being. A scoping review is an appropriate method to gather, organize, and chart the evidence [16] related to refugees living with host families. It aims to pinpoint variances and parallels in homestay hosting approaches and practices, enhancing our comprehension of their effects on refugee well-being. This review is intended to inform future research that could guide evolving practices or models for refugee hosting.

### Review Question

What is known from the existing literature about the experiences of refugees with homestay hosting?

## Methods

### Overview

The proposed scoping review will be conducted in accordance with the JBI methodology for scoping review [16] and the PRISMA-ScR (Preferred Reporting Items for Systematic and Meta-Analyses extension for Scoping Reviews) [16,17]. The protocol of this review has been registered with the Open Science Framework [18].

### Eligibility Criteria

#### Participants

This review will consider studies involving participants who identify as refugees, displaced persons, or asylum seekers. There will be no restrictions based on age or gender.

#### Concept

This review will consider literature that pertains to refugee hosting experience, hosting practices, homestay, host-guest relationship, cohabitation, and refugee well-being.

#### Context

This review will consider literature specific to refugee accommodation, hosting family, and homestay hosting practices in different host countries and different geographical settings.

#### Types of Sources

This review will consider all types of study designs. Database searches will not be limited to a particular evidence source type. Primary research articles, review articles, editorials, theses, dissertations, reports, publications after 2011, and other gray literature in English will be included. This scoping review will also include theses and conference proceedings. Opinion papers will also be considered for inclusion in this scoping review, depending on the focus and context.

### Search Strategy

In collaboration with a research librarian, the search strategy was built using some seed articles supplied to the librarian by the lead researcher as well as an initial search of several databases using basic terms. The search strategy, encompassing all identified keywords and index terms, will be tailored to suit each database and information source included. These searches were built up and used to develop a full search strategy for CINAHL (Multimedia Appendix 1). For other interfaces, the search will be modified as per the accepted Booleans, etc. The final scoping review will include all searches as appendices. The search strategy was also peer-reviewed by a fellow librarian using the PRESS Peer Review Strategy [19]. Studies published in English since 2011 will be included. The reference list of all included sources will be screened for additional studies. The databases to be searched include CINAHL, SOCIndex, MEDLINE, and Academic Search Complete through the EBSCO interface; APA PsycInfo and Scopus through the OVID interface; and Web of Science Core Collection, ProQuest Dissertations and Theses, SciELO Citation Index, and ProQuest through the Web of Science interface. As no source filters will be used, gray literature on these databases, such as conference proceedings and theses, will also be captured.

### Study Selection

Following the search, all identified citations will be collated and uploaded into EndNote (version 21; Clarivate) and duplicates will be removed. Following a pilot test, titles and abstracts will then be screened by 2 or more independent reviewers for assessment against the inclusion criteria for the review. Potentially relevant studies will be retrieved in full and their citation details imported into the JBI System for the Unified Management of the Assessment and Review of Information (SUMARI) for the unified management, assessment, and review of information [20]. The full text of selected citations will be assessed in detail against the inclusion criteria by 2 or more independent reviewers. Reasons for the exclusion of papers in full text that do not meet the inclusion criteria will be recorded and reported in an appendix to the full review. Any disagreements that arise between the reviewers at each stage of the selection process will be resolved through discussion or with an additional reviewer. The results of the search and the study inclusion process will be reported in full in the final scoping review and presented in a PRISMA (Preferred Reporting Items for Systematic and Meta-Analyses) flow diagram [16,17].

### Data Extraction

Data will be extracted from studies included in the review by 2 independent reviewers using a data extraction tool developed by the reviewers based on content relevant to the review question (Multimedia Appendix 2). The data extracted will include specific details about the participants, concept, context, study methods, and key findings relevant to the review question. The initial data extraction tool will undergo adjustments and updates as needed throughout the data extraction phase from each selected source of evidence. These changes will be thoroughly documented in the scoping review. Any disagreements that arise between the reviewers will be resolved through discussion or with a third reviewer. We will reach out to the authors for further details or clarifications when necessary. Should we encounter missing, unclear, or ambiguous data crucial for our analysis, we will contact the respective authors.

## Results

To address this scoping review’s objective of identifying, collating, and mapping the evidence about the term homestay hosting in relation to refugee groups with their host families in different host countries, we will undertake a comprehensive and thorough analytical process. This will involve synthesizing information from the selected studies into a cohesive framework. The key outcomes of this process will be systematically documented in a detailed table, which will encapsulate the diverse definitions, interpretations, and implementations of “homestay hosting” encountered in the literature (Multimedia Appendix 3). This will serve as a crucial reference point, offering a consolidated view of how “homestay hosting” is understood and applied by different researchers and practitioners in the field. Beyond merely summarizing these findings, the review will extend its analysis to include the development of diagrams. These visual aids, to be included in Multimedia Appendix 4 are designed to visually represent the relationships and connections between the various elements of refugee homestay hosting.

The diagramming effort aims to clarify how the specific aspects of homestay hosting interact with the broader concept of family hosting and with each other. This visual mapping will not only make the complex interrelations more accessible but also highlight potential areas for further research or intervention. Complementing the tables and diagrams, a narrative summary will be provided to weave together the review’s findings in a coherent format. This narrative will contextualize the evidence within the scope of the review’s objectives, offering insights into how the concept of homestay hosting contributes to our understanding of refugee support mechanisms. It will explore the implications of the findings for policy, practice, and future research, particularly emphasizing the nuances of refugee experiences in homestay settings and the roles of host families. Through this multifaceted approach—combining tabular summaries, visual diagrams, and narrative synthesis—the scoping review will offer a comprehensive and nuanced exploration of refugee homestay hosting, thereby enriching the discourse on refugee accommodation and support strategies.

## Discussion

### Overview

This scoping review will shed light on the intricate relationship between homestay hosting environments and refugee well-being, offering new perspectives on how these arrangements affect refugees’ lives. By dissecting the dynamics within homestay settings, our analysis will bring to the forefront the multifaceted ways in which these hosting arrangements influence refugee well-being. Such insights are invaluable for policy makers and practitioners tasked with crafting and executing refugee hosting programs and models, providing them with a deeper understanding of the elements that contribute to successful refugees’ integration and support. Our findings will highlight critical areas ripe for further exploration, suggesting that while this review lays the groundwork, it also underscores the necessity for more focused research. Detailed investigations, particularly longitudinal studies, could elucidate the evolving nature of refugee well-being within homestay contexts, offering a clearer picture of long-term outcomes. Similarly, comparative analyses of various hosting models could reveal best practices, guiding the development of more effective and supportive hosting frameworks. While this scoping review opens the door to a better understanding of the complex interplay between homestay hosting and refugee well-being, it also highlights the critical need for ongoing research. Future studies, armed with the foundational knowledge provided by this review, have the potential to further dissect and enhance the nuances of the homestay-refugee relationship. Such research is crucial for refining hosting practices and policies, aiming for a more empathetic and effective approach to supporting refugees worldwide, ultimately contributing to their well-being and successful integration into host communities.

### Implications

The implications of our review extend beyond academic discourse, offering actionable insights for policy makers, practitioners, and researchers alike. By advocating for tailored support mechanisms, clear policy guidelines, and further research into the long-term effects of homestay arrangements, this review sets the stage for future studies that can build on our findings. Ultimately, we hope that our work will pave the way for more empathetic, effective, and sustainable refugee support practices worldwide, ensuring that both refugees and their host families can thrive together in their shared communities.

### Limitations

This review protocol is not without limitations. One significant constraint is the scope of the literature reviewed, which may not encompass all existing studies on homestay hosting and refugee well-being due to language and publication date restrictions. Additionally, the inherent nature of scoping reviews to broadly map a field of study implies that the depth of analysis on specific topics might be limited and there will be no quality assessment of the selected resources. This limitation points to the necessity for targeted, in-depth studies that can build on the preliminary insights offered here. Furthermore, the diversity of homestay arrangements and refugee experiences across different cultural and geographical contexts may not be fully captured, highlighting the need for context-specific research to fully understand the nuances of homestay hosting and its impact on refugee well-being.

### Conclusion

This scoping review will highlight the complex relationship between homestay hosting and refugee well-being, revealing both the potential and challenges of such arrangements. By examining the current state of research, the multifaceted nature of these hosting arrangements and their effects on both refugees and their host families will be illuminated. Our findings can highlight the critical need for policies and practices that are deeply informed by an understanding of these intricate dynamics to support the well-being of refugees and their hosts effectively. Understanding the intricate dynamics of homestay hosting arrangements is crucial for developing policies and programs that support the well-being of refugees and the families that host them. This review will shed light on the current knowledge landscape, identifying research gaps and suggesting ways to enhance the homestay hosting experience for all parties involved. This review’s findings will inform the development of more inclusive, supportive, and effective approaches to refugee hosting, resettlement, and integration. To the best of our knowledge, this review represents the inaugural scoping review designed to explore the dynamic of homestay hosting and refugee well-being and the benefits such exploration brings to inform the development of refugee hosting models and practices.

## Appendix

Multimedia Appendix 1 Search strategy.

Multimedia Appendix 2 Data extraction tool.

Multimedia Appendix 3 Table of key findings.

Multimedia Appendix 4 Term map diagram.

Multimedia Appendix 5 Peer-review report from the Social Sciences and Humanities Research Council (SSHRC).

## Abbreviations

JBI: Joanna Briggs Institute
PRISMA: Preferred Reporting Items for Systematic and Meta-Analyses
PRISMA-ScR: Preferred Reporting Items for Systematic and Meta-Analyses extension for Scoping Reviews
SUMARI: System for the Unified Management of the Assessment and Review of Information

## Data Availability

This review protocol’s supporting data are shared in the manuscript appendices, enhancing transparency and understanding. These appendices include the search strategy, data extraction tool or instrument, table of key findings, and term map diagram, offering insights into our methodology, data analysis, findings, and conceptual framework. For further details or clarifications, readers are encouraged to contact the corresponding author.

## Declaration

AAH, YMY, K Metersky, and SG are registered nurses and researchers deeply committed to issues of equity and social justice in practice and inquiry and situate themselves as immigrants working to support the health and well-being of refugee groups. The work of this scoping review represents a continuation of a program of research dedicated to supporting meaningful hosting, settlement, and integration of refugee groups as a fundamental aspect of promoting and protecting their health and well-being.
