# Supplementary material for: Homestay Hosting Dynamics and Refugee Well-Being: Protocol for a Scoping Review
Source: JMIR Res Protoc. 2024 Mar 19;13:e56242. doi: 10.2196/56242 (PMC10988367; doi:10.2196/56242)
Supplement: Multimedia Appendix 1 [file resprot_v13i1e56242_app1.docx]

**Multimedia Appendix 1: Search strategy.**

Search conducted on CINAHL (EBSCO) on 13 December 2023.
AB ( Host* or (Host* N3 famil*) or homestay or cohabit* or foster* or (hospitality* N5 famil*) or accommo* )
AND

AB ( refugee* or asylum* or (displac* (person* or people*) )

AND ( impact or effect or influence or outcome or result or experience or perception or benefit or challenges )  

Language: English
Publication year: 2011-
Note: see table below for information on how the three-line search above was built up. Each search was first individually validated before being combined into a line of terms.

| Search Development | | | | | |
| --- | --- | --- | --- | --- | --- |
| Search Philosophy: find roots from within the research question What is known from the existing literature about the **experiences [search group 3]** of refugees **[search group 2]** with homestay hosting **[search group 1]**? | | | | | |
| Final search S8(abstract) AND S13(abstract) AND S23, limited to English and 2011 onwards | | | | | |
| **Search** | **Group 1 host families and similar** | **Search** | **Group 2 refugees and similar** | **Search** | **Group 3 experiences and similar** |
| S1 | Host* | S9 | Refugee* | S14 | Impact |
| S2 | Host* N3 famil* | S10 | Asylum* | S15 | Effect |
| S3 | Homestay | S11 | Displac* person* | S16 | Influence |
| S4 | Cohabit* | S12 | Displac* people* | S17 | Outcome |
| S5 | Foster* | S13 | S9 OR S10 OR S11 OR S12 | S18 | Result |
| S6 | Hospitality* N5 family* |  |  | S19 | Experience |
| S7 | Accommo* |  |  | S20 | perception |
| S8 | S1 OR S2 OR S3 OR S4 OR S5 OR S6 OR S7 |  |  | S21 | benefit |
|  |  |  |  | S22 | challenges |
|  |  |  |  | S23 | S14 OR … S22 |
