# Supplementary material for: Homestay Hosting Dynamics and Refugee Well-Being: Protocol for a Scoping Review
Source: JMIR Res Protoc. 2024 Mar 19;13:e56242. doi: 10.2196/56242 (PMC10988367; doi:10.2196/56242)
Supplement: Multimedia Appendix 2 [file resprot_v13i1e56242_app2.docx]

**Multimedia Appendix 2: Data extraction tool.**

**Peer-reviewed Literature**

| Author Year/ time frame | Country/study location | Study design | Aim/objective | Study population | Findings |
| --- | --- | --- | --- | --- | --- |

**Grey Literature**

| Author/ time frame | Organization/ Institution / study location | Study design | Focus | Study population | Key Findings |
| --- | --- | --- | --- | --- | --- |
