# Supplementary material for: Homestay Hosting Dynamics and Refugee Well-Being: Protocol for a Scoping Review
Source: JMIR Res Protoc. 2024 Mar 19;13:e56242. doi: 10.2196/56242 (PMC10988367; doi:10.2196/56242)
Supplement: Multimedia Appendix 3 [file resprot_v13i1e56242_app3.docx]

**Multimedia Appendix 3: Table of key findings.**

| Author, year, study location | Study design | Aim/objective | Setting/ Study population | Application of term refugee homestay hosting |
| --- | --- | --- | --- | --- |
