# Supplementary figures and images for: Homestay Hosting Dynamics and Refugee Well-Being: Protocol for a Scoping Review
Source: JMIR Res Protoc. 2024 Mar 19;13:e56242. doi: 10.2196/56242 (PMC10988367; doi:10.2196/56242)

**Multimedia Appendix 4: Term map diagram.**


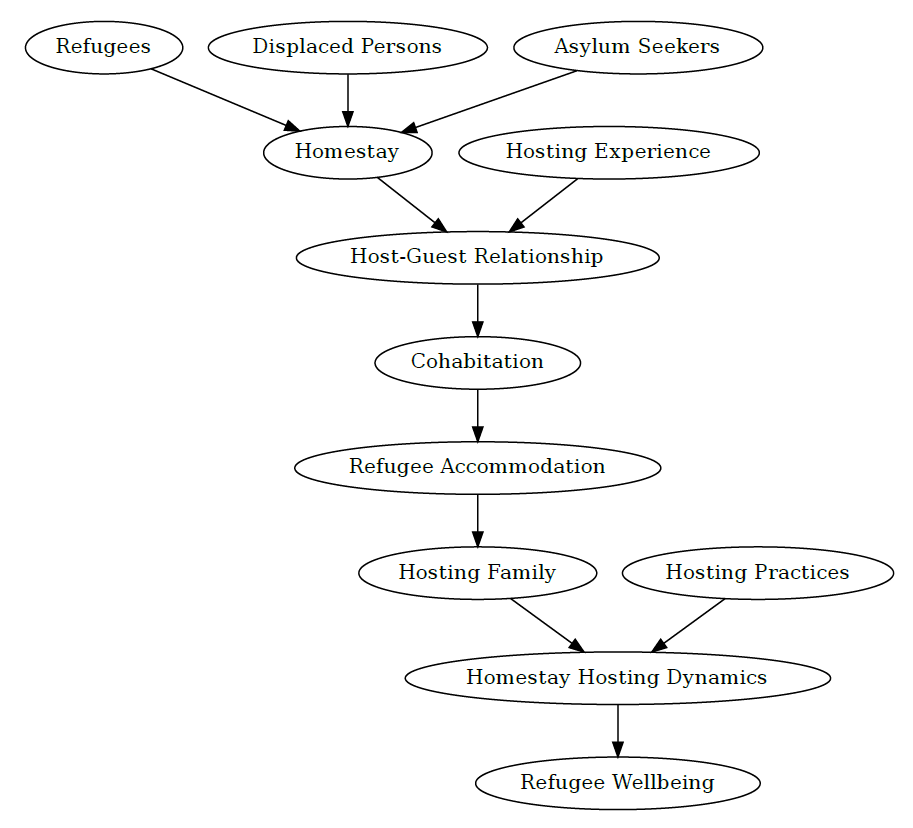

Supplement: Multimedia Appendix 4 [file resprot_v13i1e56242_app4.docx]
