# Supplementary material for: Homestay Hosting Dynamics and Refugee Well-Being: Protocol for a Scoping Review
Source: JMIR Res Protoc. 2024 Mar 19;13:e56242. doi: 10.2196/56242 (PMC10988367; doi:10.2196/56242)
Supplement: Multimedia Appendix 5 [file resprot_v13i1e56242_app5.pdf]

Insight Development Grants Committee Evaluation Form

**Committee:**	430-16A - Communications, media studies, gender studies, library and information science, related fields
**Application ID:**	430-2023-00080
**Applicant Name:**	Areej Al-Hamad
**Application Title:**	Picturing the experiences of refugees: A Photovoice exploration of Ukrainian refugee women's experiences with their Canadian hosts in the Greater Toronto Area
**Scholar Type:**	Emerging

Part 1: Challenge - The aim and importance of the endeavour (50%)

Sub-criteria (No specific weighting assigned to each sub-criterion)	N/A	Unsatisfactory	Moderate	Satisfactory	Good	Very Good	Excellent
For established scholars: the proposal's relevance to the objectives of the funding opportunity	X						
Originality, significance and expected contribution to knowledge						X	
Appropriateness of the literature review						X	
Appropriateness of the theoretical approach or framework						X	
Appropriateness of the methods/approach				X			
Quality of training and mentoring to be provided to students, emerging scholars and other highly qualified personnel, and opportunities for them to contribute						X	
Potential for the project results to have influence and impact within and/or beyond the social sciences and humanities research community						X	

For questions, please contact us at: insightdevelopment@sshrc-crsh.gc.ca

Part 2: Feasibility - The plan to achieve excellence (20%)

Sub-criteria (No specific weighting assigned to each sub-criterion)	N/A	Unsatisfactory	Moderate	Satisfactory	Good	Very Good	Excellent
Appropriateness of the proposed timeline and probability that the objectives will be met					X		
Expertise of the applicant or team in relation to the proposed research				X			
Appropriateness of the requested budget, justification of proposed costs, and, where applicable, other financial and/or in-kind contributions				X			
Quality and appropriateness of knowledge mobilization plans, including for effective dissemination, exchange and engagement with stakeholders within and/or beyond the research community, where applicable					X		

Part 3: Capability - The expertise to succeed (30%)

Sub-criteria (No specific weighting assigned to each sub-criterion)	N/A	Unsatisfactory	Moderate	Satisfactory	Good	Very Good	Excellent
Quality, quantity and significance of past experience and published and/or creative outputs of the applicant and any co-applicants, relative to their roles in the project and to the stage of their career					X		
Evidence of past knowledge mobilization activities (e.g. films, performances, commissioned reports, knowledge syntheses, experience in collaboration/other interactions with stakeholders, contributions to public debate and media), and of impacts on professional practice, social services and policies, etc.					X		
Quality and quantity of past contributions to the development of effective research training and mentoring of students, postdoctoral researchers and other highly qualified personnel					X		

For questions, please contact us at: insightdevelopment@sshrc-crsh.gc.ca

Part 4: Comments

The committee expressed concern regarding the anonymity of the participants and the power relations of the proposed research. It would have liked to see the proposal address how the participants would be protected. Furthermore, it found the compensation for participants to be low considering the emotional labour required for the project.

The committee found that the knowledge mobilization plan required more development. It noted that the proposed program seemed to overly depend on the university to disseminate information about the study.

The committee noted with approval that the applicant has done similar work in the past. However, it considered that the low participation of the co-applicants may be a barrier to the feasibility of the project.
